# A Staged Defragmented Simultaneous Debriefing Model As Integrated Micro-debriefing Components Inside Online Simulation for Competencies Formation

**DOI:** 10.7759/cureus.56000

**Published:** 2024-03-12

**Authors:** Nataliia Lopina

**Affiliations:** 1 Simulation Training Platform, ClinCaseQuest, Kharkiv, UKR

**Keywords:** debriefing in action, reflective pause, competency-based learning, medical errors, competency formation, online clinical simulation, simultaneous debriefing, defragmented debriefing, micro-debriefing, debriefing

## Abstract

Background

Currently, there are no separate debriefing models for online simulation training, and existing models simply imitate the traditional models used in on-site simulation training (the physical presence of individuals, such as students or trainees, in a simulation center). This involves hands-on, in-person training within a simulated environment to enhance practical skills and knowledge in a controlled setting. This scenario does not fully meet the requirements and capabilities of distance learning.

Objective

To develop a staged defragmented debriefing model as integrated micro-debriefing components inside an online simulation to support the development of clinical decision-making and competencies formation within medical education and offer recommendations to support the use of this debriefing model as a teaching strategy.

Methods

This descriptive study was conducted from August 2020 to September 2023. To build a staged defragmented debriefing model as integrated micro-debriefing components inside an online simulation for competencies formation the traditional debriefing model's components for on-site simulation training, simulation type, and structure, modern concepts of e-learning, and classification of the seriousness of medication errors were used. The main focus of this study was on providing a detailed account of the debriefing components for online simulation training, features, and implementation of this new teaching model. A total of 38 participants, healthcare professionals, were recruited for this study. The participants were randomly assigned to two groups: one experiencing the staged defragmented debriefing model (n = 20) and the other control group, which received traditional debriefing following simulation training (n = 18).

Results

The results allowed us to successfully develop a staged defragmented debriefing model inside the simulation that integrates micro-debriefing components located at different points of the simulation scenarios. This teaching approach was successfully implemented in online clinical case scenarios in the “ClinCaseQuest" Simulation Training Platform for continuous medical education. Additionally, an internal validation experiment comparing the effectiveness of the staged defragmented debriefing model with the traditional debriefing method demonstrated superior learning outcomes and participant satisfaction in the staged debriefing group.

Conclusions

The staged defragmented debriefing model, when integrated into online simulations, represents a promising strategy for advancing clinical decision-making skills and competencies formation in medical education. Implementation of this debriefing model as a teaching strategy holds promise for enhancing learning outcomes in medical education settings. Further research, validation, and implementation are recommended to maximize the model’s potential impact on medical education and training.

## Introduction

Online clinical simulation creates a virtual environment that replicates various healthcare settings such as hospitals, clinics, or emergency rooms [1]. This environment can be accessed through web-based platforms or specialized software [2]. Educators design interactive scenarios that mimic real-life patient encounters and can involve medical history taking, physical examinations, diagnostic decision-making, treatment planning, and communication with patients or colleagues [3-5].

Online clinical simulation provides several opportunities to improve aspects of healthcare education and training such as accessibility, scalability, cost-effectiveness, safe learning environment, standardized training, enhanced feedback and assessment, collaborative learning, lifelong learning, and continuing education [3].

Overall, online clinical simulation opens up new possibilities for healthcare education and training, providing flexible, accessible, and immersive learning experiences that can improve clinical competency, patient safety, and overall healthcare outcomes [5].

Debriefing following a simulation is widely recognized as one of the most impactful elements of Simulation-Based Education (SBE) and serves as a fundamental pillar in the educational process in medical simulation environments [6,7].

Debriefing entails purposeful discussions conducted after a simulation experience using reflection, facilitating participants' comprehension of their actions and cognitive processes to foster learning outcomes and elevate subsequent clinical performance. It provides an opportunity for both educators and learners to revisit the simulated case encounter, refine their cognitive frameworks, and cultivate the rationale behind their clinical decision-making [8].

Kolb’s experiential learning cycle begins with a tangible encounter, often in the form of a simulation. Participants then engage in observation and contemplation of this experience during the debriefing phase. According to experiential learning theory, this sequence contributes to the development of abstract concepts and overarching principles, which are subsequently applied to test hypotheses in future situations and culminate in new, real-world experiences. The reflective component of debriefing plays a pivotal role in anchoring experiential learning theory [9,10].

Standardized simulation training including briefing, scenario execution, debriefing, feedback, and reflection, collectively form a widely acknowledged and established framework. Simulation training also should include standardized debriefing models. The effectiveness of debriefing currently hinges on the session leader’s level of training and expertise [11,12]. Online clinical simulation gives many opportunities for feedback and guidance in real-time, including immediate feedback on clinical decisions, performance metrics, and suggestions for improvement. Facilitators or virtual coaches may also be available to guide learners through the scenarios.

Currently, there are no separate debriefing models for online simulation training, and existing models merely imitate the traditional models used in on-site simulation training, which does not fully meet the requirements and capabilities of distance learning. Online simulations present a distinct set of challenges and opportunities that demand a specialized debriefing methodology. Unlike in-person on-site simulation training, online environments necessitate a reevaluation of debriefing strategies to account for factors such as:

- asynchronous nature of the interaction in online simulation;

- potential technological limitations for online simulation training;

- the need for increased adaptability of medical simulation;

- the need to foster participant engagement, maintain a sense of immersion, and ensure effective communication inside the simulation.

Conventional debriefing models may not adequately address these nuances, leading to a potential disconnect between the debriefing process and the online learning environment. The separate debriefing approach is not merely a call for novelty but stems from the recognition that the distinctive characteristics of online simulations necessitate tailored strategies to optimize learning outcomes. Developing a dedicated debriefing model for online simulations fills a gap in the current literature and provides educators and practitioners with a valuable resource that aligns with the unique demands of distance learning. In essence, the proposed debriefing approach for online simulations seeks to enhance the effectiveness of the debriefing process by acknowledging and addressing the specific challenges and opportunities presented by the online learning environment, ultimately contributing to the advancement of best practices in simulation-based distance education.

The effectiveness of debriefing is closely tied to the expertise and training of the debriefer. Unfortunately, there can be variations in the level of training among debriefers, and this can impact the quality of the educational experience for learners. The consistency in learning objectives is connected with the debriefing strategy effectiveness in the notion that, despite potential differences in debriefers' experience and training, learners should ideally receive a good quality educational experience.

Efforts should be made to ensure that differences in debriefers' expertise do not compromise the overall educational outcome. This ongoing work seeks to explore ways to mitigate the impact of varying debriefer experiences, ensuring a consistently high-quality educational experience for learners across different settings.

Considering the diverse factors influencing simulation experiences, it is imperative to refine the debriefing process. The paper introduces a novel debriefing model, emphasizing its innovative approach to distinguish it from existing models. This approach aims to establish a unique and tailored educational experience while ensuring a consistent and uniform standard across different locations and institutions.

Simulation experiences can vary based on learner actions, and the learning objectives should be met, even if the paths to achieving them differ. This work recognized the dynamic nature of simulation scenarios and the diverse ways in which learners may approach and respond to these experiences. To address this variability and ensure that all learners meet the intended learning objectives, an adaptive debriefing model was developed. This adaptive debriefing model format is based on learners' actions during the simulation. This approach recognizes the diversity in learner actions and pathways to achieving the learning objectives while maintaining a focus on meeting those objectives consistently.

The article presented a teaching strategy for online debriefing scripts to prepare for online clinical simulation as a staged defragmented debriefing model with integrated micro-debriefing components.

In the context of this work, "staged debriefing" refers to a structured and sequenced approach to the debriefing process divided into parts. Rather than a single, continuous debriefing session, the described approach proposes breaking it into distinct stages to allow for a focused discussion on specific aspects of the simulation. In simulation training, each part serves a distinct purpose, and errors from each stage should not be accumulated or carried to the simulation's conclusion. The quality of the training will be very low. We work through simulation in parts, including integrating a step-by-step debriefing strategy. This aims to avoid information overload and enhance the clarity and effectiveness of each debriefing phase as well as each part of simulation training.

"Embedded micro-debriefing components" refer to the incorporation of smaller, targeted debriefing elements within the simulation training. These micro-debriefing components are seamlessly woven into the overall simulation training, addressing specific aspects of the simulation experience.

In essence, this teaching strategy for online debriefing scripts involves a methodical and structured approach to the debriefing process with fragmentation to enhance the results. Within this framework, micro-debriefing components were integrated into the simulation. This strategy aims to provide a clear, organized, and comprehensive debriefing experience for online clinical simulations.

## Materials and methods

This study was conducted from August 2020 to September 2023. The study was conducted at the Simulation Training Platform for continuous medical education "ClinCaseQuest" аs internal validation experiment. The research aimed to develop a staged defragmented simultaneous debriefing model as integrated micro-debriefing components inside online simulation for competencies formation, describe its components, and provide the validation experiment aimed to compare the effectiveness of the proposed staged defragmented debriefing model with traditional debriefing method commonly used in simulation-based medical education based on learning outcomes, participant satisfaction, and overall effectiveness in the context of online simulation training.

The following search terms and concepts and their Boolean combinations to conduct a literature search based on PubMed, Cochrane Library, ERIC, and PsycINFO were employed: debriefing, online debriefing, debriefing models, and virtual and online simulation. The search revealed some proposals for providing online debriefing; however, these proposals simulated debriefing models for on-site simulation training in the majority of cases [13-19]. In the described models of online debriefing, the student depends on the trainer and the training group.

This new model of debriefing is based on:

1. traditional debriefing model components for on-site simulation training [20-28];

2. simulation type and components, structure of the simulation training (based on the author's point of view);

3. modern concepts of e-learning [29-31];

4. classification of the seriousness of medication errors [32].

The next components came from on-site simulation training debriefing sessions (Plus-Delta; Reaction, Analysis, and summary (RAS); Gather, Analysis, Summarize (GAS); Defusing, discovering, and deepening (3D Model); Debriefing in Medical Education DIAMOND; Promoting Excellence And Reflective Learning in Simulation (PEARL TEAM); Hybrid, structured debriefing tool for simulation-based team training in healthcare (TEAM GAINS); After Action Review (AAR)) (Table 1) [20-28].

**Table 1:** The main components of the on-site simulation training debriefing sessions are based on available debriefing models.

Components of the on-site simulation training debriefing sessions	Abbreviation for further model developed
Gather	g
Description phase	d
Reaction	r
Discuss clinical component	dcc
Observe the patient's state	o
Analysis (with deepening)	a
Summary	s
Application / Transfer to reality	a/t

An “Observe the patient's state” component for the patient’s observation during the simulation was included. A learner could observe a patient’s condition getting worse or improving, which is a specific type of feedback that could be given during simulation. This component is included to emphasize the learner's role in assessing and interpreting changes in the patient's condition based on their actions during the simulation. The intention is to highlight the dynamic nature of the simulation scenario.

Traditional debriefing model components for in-site simulation training encompass established debriefing practices used in traditional, in-person on-site simulation training. These include techniques for facilitating reflection, discussing actions and decisions, and promoting a constructive learning environment. The rationale for incorporating these components inside the proposed debriefing model is to leverage proven strategies that have demonstrated effectiveness in enhancing learning outcomes during debriefing sessions, their widespread recognition in the simulation and healthcare education literature.

Simulation type and components, and structure of the simulation training should also take into account the specific type of simulation being used, along with its components and the overall structure of the simulation training. This recognizes that different simulation types may require tailored debriefing approaches. The rationale is to align the debriefing process with the unique characteristics of the simulation, ensuring that the debriefing model is contextualized and relevant to the simulated scenario.

Simulation training can serve various purposes, including competency development, assessment, or a combination of both.

Two fundamental concepts in modern e-learning are adaptive learning and microlearning. Adaptive learning is an approach that customizes the learning experience for each individual, tailoring content and pacing to the learner's abilities and needs [29,30]. Microlearning, by contrast, involves delivering content in small, focused units, typically designed to be consumed in a short amount of time. It is characterized by its brevity and effectiveness in delivering specific pieces of information or skills [31].

This integrates contemporary e-learning principles, including interactive engagement, and adaptive learning. The rationale for integration in the presented debriefing model is to leverage the benefits of modern e-learning concepts to enhance the online debriefing experience, making it more engaging, accessible, and effective for participants.

In the proposed debriefing model, adaptive learning principles are implemented by tailoring the debriefing experience to each individual learner. This customization involves adjusting the content and pacing of the debriefing session based on the learner's abilities and specific needs. For example, feedback and reflection points may be personalized to address individual learning goals or areas that require further development. The adaptive nature of the proposed debriefing model ensures a tailored and effective learning experience for each participant. Microlearning is integrated into the proposed model by delivering content in small, focused units during the debriefing process. Instead of overwhelming learners with extensive information, the presented approach strategically breaks down the debriefing content into concise, digestible segments. This approach aligns with the brevity and effectiveness characteristic of microlearning, allowing learners to absorb specific pieces of information or skills in a short amount of time. Together, these concepts contribute to the efficiency and learner-centric nature of this debriefing model. Personalizing the experience and delivering information in a targeted manner, could enhance the overall effectiveness and engagement of the debriefing process for online simulation training.

A medical error was chosen as a potential point for integration components of defragmented debriefing.

Classification of the seriousness of medication errors was determined according to the National Coordinating Council for Medication Error Reporting and Prevention (NCCMERP) (Table 2) [32].

**Table 2:** Classification of the seriousness of medication errors according to the National Coordinating Council for Medication Error Reporting and Prevention (NCCMERP). Harm: impairment of the physical, emotional, or psychological function or structure of the body and/or pain resulting therefrom. Monitoring: observing or recording relevant physiological or psychological signs. Medical Intervention: change in therapy or active medical or surgical treatment. Vital Support Intervention: cardiovascular and respiratory support (e.g., CPR, defibrillation, intubation, etc.). Permission was granted by the National Coordinating Council for Medication Error Reporting and Prevention [32].

Error category	Error occurrence	Reached patient	Associated harm	Necessary measures
A	Potential	No	No	No
B	Yes	No	No	No
C	Yes	Yes	No	No
D	Yes	Yes	No	Monitoring
E	Yes	Yes	Temporary	Medical intervention
F	Yes	Yes	Temporary	Hospitalization or prolonged hospital stay
G	Yes	Yes	Permanent	Variable
H	Yes	Yes	Risk of death	Vital support intervention
I	Yes	Yes	Death	-

**Figure 1:**
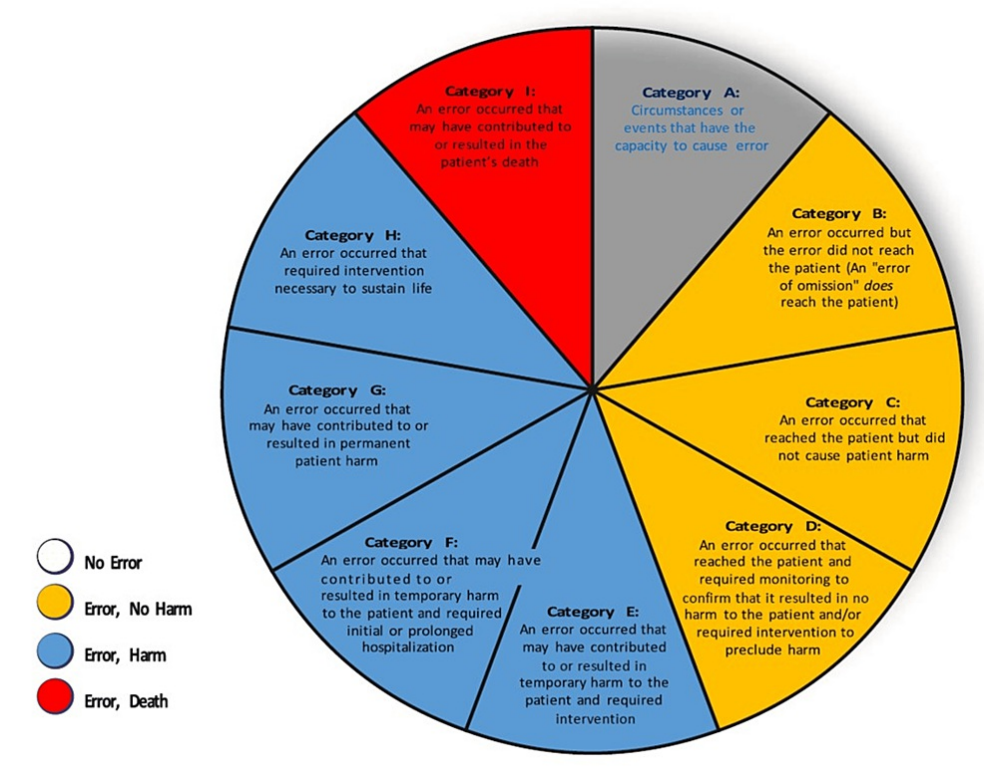
Classification of the seriousness of medication errors according to the National Coordinating Council for Medication Error Reporting and Prevention (NCCMERP). Permission was granted by the National Coordinating Council for Medication Error Reporting and Prevention.

There are nine different categories for seriousness from A to I. Each classification includes the occurrence of an error, whether the error reached the patient, the harm associated with the error, and the necessary measures (Figure 1) [32].

This system could be useful in preparing clinical case scenario scripts in adaptive learning formats for online simulation for integration of micro-components of debriefing inside simulation.

By incorporating this classification, the debriefing model aims to provide targeted feedback and reflection points based on the severity of medical errors in general (not only connected with drug usage), contributing to a more focused and impactful debriefing process.

The decision to choose a medical error as a potential point for integration in the defragmented debriefing model is rooted in the strategic goal of enhancing the effectiveness of competency development. Medical errors, occurring at various levels of severity, serve as critical junctures within the simulated clinical scenario. It is at these points that the development of the clinical situation can dynamically change. By selecting medical errors as integration components, the aim is to capitalize on these pivotal moments to provide targeted feedback to students. The severity and type of medical error become catalysts for discussions that enable a deeper exploration of decision-making, critical thinking, and clinical reasoning. This approach ensures that learners receive focused and constructive feedback, facilitating a more impactful reflection on their actions and decisions during the simulation. In essence, the choice of integrating medical errors aligns overarching goal of maximizing the effectiveness of the debriefing process in developing competencies. It leverages these critical incidents to offer valuable insights and learning opportunities, contributing to a more robust and targeted debriefing experience for participants.

The classification (National Coordinating Council for Medication Error Reporting and Prevention (NCCMERP)) fully caters to the needs of simulation education. It offers the flexibility to modify the patient's condition and stratifies medical errors by severity, facilitating correlation with scoring systems when calculating students' traditional performance indicators (GPAs) and grades.

This assessment system is founded on the stratification of medical errors according to their severity, the extent of harm inflicted on the patient, and an evaluation of the measures required to address the consequences of the error. This aligns ideally with the game mechanics employed in constructing virtual, interactive scenarios of clinical cases. Depending on the actions of the learner, the scenario's development and the provided information may change, allowing for the creation of an adaptive learning format. Introducing micro-components of debriefing within the simulation can vary based on whether trainees make specific medical errors of varying severity. This system of medical errors provides a convenient means to predict the development of a clinical situation, taking into account changes in the patient's condition and the necessary assistance. This classification system facilitates the scoring of simulation training.

The combination of the presented components of the proposed debriefing model is not intended to be additive in a mathematical sense. Instead, each component brings a unique contribution to the overall debriefing model, and their combination is designed to create a comprehensive, adaptable, and effective approach to online debriefing.

This article provides a detailed exploration of how these components interact synergistically, offering a nuanced and context-specific debriefing model for online simulation training.

The experiment employed a randomized controlled trial design, with participants randomly assigned to either the experimental group, which experienced the staged defragmented debriefing model, or the control group, which received traditional debriefing following simulation training in a standard online web conference with the instructor. A traditional debriefing model was chosen (RAS model - Reaction, Analysis, Summary).

A total of 38 participants, healthcare professionals, were recruited for this study. The participants were randomly assigned to two groups: one experiencing the staged defragmented debriefing model (n = 20) and the other control group, which received traditional debriefing following simulation training (n = 18). The inclusion criteria for participation in the study comprised healthcare professionals who voluntarily consented to take part. There were no additional criteria for inclusion or exclusion. The participants were recruited from a diverse range of healthcare institutions, including hospitals, clinics, and medical centers, spanning different regions of Ukraine after self-registration on the platform.

Participants in both groups underwent identical simulation case training. The experimental group experienced the staged defragmented debriefing model, characterized by structured debriefing stages and integrated micro-debriefing components, while the control group received traditional debriefing led by an instructor following the completion of the simulation training.

Data were collected through a combination of quantitative surveys and qualitative feedback forms administered to participants immediately following the debriefing sessions.

Learning outcomes, participant satisfaction, and overall effectiveness were assessed. Each component is assessed using a 5-point Likert scale, with mean values and standard deviations provided for each group. Higher mean values indicate higher levels of effectiveness or satisfaction.

Quantitative data were analyzed using statistical methods, including descriptive statistics, and t-tests to compare outcomes between the experimental and control groups.

The study was conducted following ethical principles, including those outlined in the Declaration of Helsinki, and informed consent was obtained from all participants.

## Results

A staged defragmented debriefing model for integrated micro-debriefing components in an online simulation of competencies formation was developed.

Staged defragmented simultaneous debriefing is a demonstration of the consequences of the decision-making process, emotional involvement, reasoning, explanations, cognitive support, discussions, and immediate feedback in simulation training. Learners receive simultaneous simulation training in a synchronous format depending on their actions in accordance with the classification of the seriousness of medication errors as an adaptive learning strategy with microlearning components. In online simulation training, micro-debriefing points are determined based on the actions of students during the simulation. Depending on the actions taken by the learner, the system selects the appropriate scenario to further develop the clinical situation. Micro-debriefing components in this context are designed to be more specific and targeted than traditional feedback. While feedback may be general, micro-debriefing is a focused discussion or reflection on specific aspects of the learner's actions. It allows for a deeper exploration of decision-making, encouraging learners to reflect on their choices and understand the implications in the context of medical errors. In essence, the use of branching scripts enables the system to tailor the simulation experience for each learner, providing targeted micro-debriefing components where needed. This approach enhances the adaptability and effectiveness of the training, ensuring a personalized and impactful learning experience for participants.

If learners avoid making errors, it is possible to incorporate a micro-debriefing component to reinforce the correct choice or provide feedback with approval and support as necessary.

A debriefing strategy based on traditional components of on-site simulation training debriefing sessions was presented. The meaning of the debriefing component if performed as an online simulation and proposed corresponding micro-debriefing components for integration inside online simulation was expanded (Table 3).

**Table 3:** Micro-debriefing components inside an online simulation based on the components of available debriefing models in on-site simulation training. a: analysis (with deepening); a/t: application and transfer to reality; g: gather; d: description phase; r: reaction and report; dcc: discuss clinical component; o: observe the patient's state; s: summary

Components of the on-site simulation training debriefing sessions	Abbreviation for further model developed	Meaning of the debriefing component in online simulation	Micro-debriefing components inside an online simulation
Gather	g	Gathering learner’s opinions and feedback after simulation training	Filling out the feedback form after the simulation training, board or joint chat, or group webinar
Description Phase	d	This phase focuses on the factual description of events and activities that took place during the simulation. Participants share their observations, highlighting key points, decisions, and interactions. This step helps establish a common understanding of the scenario.	Filling out the feedback form after the simulation training, board or joint chat, or group webinar
Reaction and Report	r	Receiving learners’ reactions and after simulation training, analyzing learners' reactions inside the simulation based on online simulation training report	Filling out the feedback form after the simulation training, board, or joint chat. Analyzing learners’ reactions inside simulation based on online simulation training report
Discuss clinical component	dcc	Immediate feedback during online simulation from virtual supervisors and colleagues	Short explanations, short comments on positive and negative learner’s actions (support for correct decision-making, hint for incorrect actions, or additional guiding questions that can help learners get through a difficult part of the scenario) integrated inside the scenario and given to the learner in adaptive learning format depending on the learner's choice, cognitive support if necessary in microformat as text, links, short videos, audios, images, quizzes, dialogue simulators integrated inside simulation training.
Observe the patient's state	o	Observe a patient’s condition getting worse or improving	Changes in patient’s state: patient’s condition getting worse or improved.
Analysis (with deepening)	a	Differential diagnosis making, preliminary diagnosis making, clinical diagnosis making, prescribing therapy	Tasks integrated into simulation for providing an analysis (with deepening) on differential diagnosis making, preliminary diagnosis making, clinical diagnosis making, prescribing therapy with feedback (comments and explanation), cognitive support if necessary in microformat as integrated text, links, short videos, images, audios, quizzes, dialogue simulators integrated inside simulation training.
Summary	s	Summary clinical case scenario gained competencies, simulation training aims at the end of clinical case simulation	Discussion of the particular clinical case scenario or discussing and modeling different developments of events in simulation in the form of explanation and storytelling as text, videos, images, audio, or dialogue simulator.
Application and Transfer to Reality	a/t	Making a bridge to real clinical practice at the end of clinical case simulation	Discussion of the most typical medical errors in real clinical practice devoted to a particular clinical topic in the form of explanation and storytelling as text, videos, images, audio.

Learners' reactions during the simulation are not collected through direct interruption or explicit feedback but are assessed indirectly. The sequence of actions performed by learners and the correctness of their decision-making serve as key indicators of their reactions. This allows for a non-disruptive and seamless flow of the simulation, preserving the authenticity of learners' responses without introducing interruptions. Post-simulation, additional reactions are collected by utilizing feedback forms. Learners are provided with an opportunity to reflect on their experiences, express their thoughts, and provide feedback through the feedback forms. This method allows for a more explicit understanding of learners' reactions, complementing the indirect assessment during the simulation. In summary, the dual approach involves both indirect assessment during continuous simulation training and explicit feedback collection through post-simulation feedback forms. This combined methodology provides a comprehensive understanding of learners' reactions without disrupting the natural flow of the simulation, ensuring a holistic evaluation of their experiences and responses.

The feedback form utilized after the simulation training is designed to capture a comprehensive range of insights from learners. The questions within the feedback form are structured to gather both specific feedback on the simulation itself and insights into learners' feelings and experiences during and after the simulation. The form includes a combination of general questions to evaluate the overall effectiveness of the simulation, as well as specific inquiries aimed at understanding learners' emotions, reactions, and reflections during different phases of the simulation. Examples of questions may include inquiries about the perceived realism of the scenario, the clarity of communication, the effectiveness of decision-making processes, and the emotional impact of the simulation experience. In essence, the feedback form serves as a multifaceted tool, allowing us to gather nuanced feedback on various aspects of the simulation, including both objective evaluations and subjective experiences. This approach ensures a comprehensive understanding of learners' reactions, contributing to a more holistic analysis of the simulation training.

During simulation training, trainees can actively witness or perceive the consequences of their decisions by observing the improvement or deterioration of the patient’s condition. The term 'observe' is chosen to emphasize the active and intentional nature of the trainees' engagement with the simulated scenario, allowing them to visually assess the impact of their actions on the patient's well-being. Also, this observation stage is connected with further steps in decision-making and reaction.

Classification of the seriousness of medication errors according to the National Coordinating Council for Medication Error Reporting and Prevention (NCCMERP) was used for the integration of micro-debriefing components inside an online simulation (Table 4) [32].

**Table 4:** Prediction draft for the simulation scenario script for integration of micro-debriefing components inside online simulation based on classification of the seriousness of medication errors according to the National Coordinating Council for Medication Error Reporting and Prevention (NCCMERP). a: analysis (with deepening); dcc: discuss clinical component; o: observe the patient's state; r: reaction and report; s: summary. Permission was granted by the National Coordinating Council for Medication Error Reporting and Prevention [32].

Error category	Error occurrence	Reached patient	Associated harm	Necessary measures	Clinical case situation development	Micro-debriefing component for integration inside the simulation
A	Potential	No	No	No	Continue	Consider debriefing intervention
B	Yes	No	No	No	Continue	Debriefing intervention in the form of r_, _dcc
C	Yes	Yes	No	No	Continue	Debriefing intervention in the form of r, dcc
D	Yes	Yes	No	Monitoring	Continue with measures for patient monitoring	Debriefing intervention in the form of r, dcc, o, a, s
E	Yes	Yes	Temporary	Medical intervention	Continue with medical intervention (if the medical intervention is appropriate, the patient's state improves, if not – use F-I classification – the patient's state getting worse and requires further steps, providing additional calculations)	Debriefing intervention in the form of r, dcc, o, a, s
F	Yes	Yes	Temporary	Hospitalization or prolonged hospital stay	Continue with medical intervention (if the medical intervention is appropriate, the patient’s state is improving, if not – use F-I classification – the patient's state getting worse and requires further steps, providing additional calculations)	Debriefing intervention in the form of r, dcc, o, a, s
G	Yes	Yes	Permanent	Variable	Continue with further decision-making steps with the possibility of clinical case termination (if further steps are appropriate, the patient's state improves, if not – use G-I classification – the patient's state getting worse and requires further steps, providing additional calculations)	Debriefing intervention in the form of r, dcc, o, a, s
H	Yes	Yes	Risk of death	Vital support intervention	Continue with further decision-making steps with the possibility of clinical case termination (if further steps are appropriate, the patient's state improves, if not – use H-I classification – the patient's state getting worse and requires further steps, providing additional calculations)	Debriefing intervention in the form of r, dcc, o, a, s
I	Yes	Yes	Death	-	Clinical scenario stop	Debriefing intervention in the form of r, dcc, o, a, s

This table provides a clearer illustration of the game mechanics and the potential development of the clinical situation. Additionally, this approach helps outline possible integration options with points of debriefing components and their types, combining this information with the classification of the severity of medical errors. This is the practical approach that is employed when creating online simulation training.

Debriefing inside a simulation (debriefing in action, reflective pause) refers to the practice of conducting debriefing during a simulation scenario as numerous small components (micro-pieces, micro-learning, micro-debriefing) integrated inside a simulation while participants are still actively engaged in the simulation environment. This approach allows for immediate real-time feedback and reflection, maximizing the learning experience and promoting the immediate application of insights, which could enhance competency formation.

During debriefing inside simulation using online tools, there are many opportunities not to interrupt a simulation training. The learner understands and feels support facilitation and guided simulation.

Micro-debriefing gives us a great opportunity for competency formation. Short debriefing inside the simulation focuses on immediate feedback and reflection on specific aspects of the scenario or performance and allows for quick course corrections and adjustments. This protocol makes it possible to avoid many medical errors and misunderstandings and not to transfer them to the end of the simulation training. By not letting errors accumulate, quick debriefing has important implications for simulation training in complex clinical scenarios with multiple clinical decisions within the multilocation simulation.

The differences from traditional debriefing are related to the methodology of the debriefing, the format of the debriefing, the change in the position of the debriefing during simulation, and the breakdown of the entire debriefing into parts.

The entire simulation training can be divided into logical blocks:

1. Depends on the components of the patient’s care (communication with a patient, objective examination, laboratory data, diagnostic tests, differential diagnosis, making a diagnosis, prescribing therapy, monitoring, and follow-up).

2. Depends on the components of the patient’s way in particular nosology (for example, patient's way with acute myocardial infarction-paramedic stage, catheter laboratory, intensive care unit, general ward).

**Figure 2:**
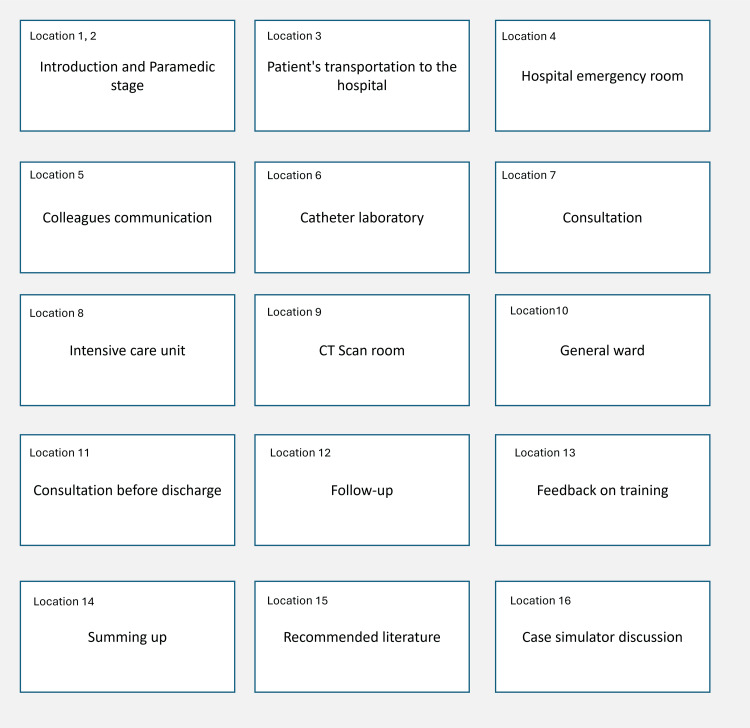
Acute myocardial infarction with ST-segment elevation in a patient with SCAD (Patient's way in the healthcare system and the structure of the serious game simulation training). CT: computer tomography; SCAD: spontaneous coronary artery dissection

Each block has different aims and a list of acquired competencies. These blocks are titled Location 1, Location 2, Location n or A, B, C, D, E, F (Figure 2).

The general structure of the simulation training is a combination of logical blocks with micro-debriefing components.

A + B + C + D + E + F+….. + Z

When planning a debriefing in parts, it is necessary to clearly define the positions and types of debriefing components:

A dcc1, dcc2, a1 + B o «+», o «-» + C dcc3, dcc4, a1 + D dcc5, a2 + E a3, a4 + F dcc6, a5 +….. + Z s1, a/t1, g1, d1, r1

A practical example of this structure is presented in Table 5.

**Table 5:** Comparison of learning outcomes, participant satisfaction, and overall effectiveness between staged defragmented debriefing and traditional debriefing models (n = 20 staged defragmented debriefing group; n = 18 for traditional debriefing following simulation training) Statistical significance was determined using a t-test to compare the means of the two groups. p-values are reported to indicate the significance of the differences observed between the groups.

Criteria	Staged defragmented debriefing group (n = 20)	Control group (traditional debriefing following simulation training) (n = 18)	p-value
Learning Outcomes			
Clinical Decision-Making Skills	4.15 ± 0.59	3.55 ± 0.51	0.002
Competencies Formation	4.3 ± 0.47	3.55 ± 0.51	<0.001
Knowledge Retention	4.35 ± 0.49	3.44 ± 0.51	<0.001
Participant Satisfaction			
Feedback Quality	4.50 ± 0.51	4.11 ± 0.58	0.03
Engagement	4.40 ± 0.50	4.83 ± 0.38	0.005
Relevance	4.35 ± 0.49	4.61 ± 0.50	>0.05
Overall Effectiveness			
Learning Experience	4.45 ± 0.51	4.05 ± 0.64	0.04
Impact on Clinical Practice	4.20 ± 0.62	4.00 ± 0.48	>0.05
Comparison with Expectations	4.20 ± 0.41	4.05 ± 0.73	>0.05

It is also possible to indicate the format of the debriefing in a particular position and the condition for performing the debriefing component (adaptive training format):

A dcc1dialogue simulator, dcc2quiz, a1 + B o «+» text (if patient‘s condition improves), o «-» video (if patient‘s condition getting worse) + C dcc3link, dcc4text, a1dialogue simulators + D dcc5text, a2quiz + E a3dialogue simulator, a4text + F dcc6dialogue simulator, a5quiz +….. + Z s1video, a/t1image, g1, d1, r1text.

The following are recommendations supporting the use of a staged defragmented debriefing model as integrated micro-debriefing components inside online simulation for competencies formation as a teaching strategy:

1. Most debriefing material could be defragmented into logical parts following the training objectives and the list of competencies to be mastered in the format of micro-components.

2. To prepare micro-components of debriefing for online training, any of the generally accepted models of debriefing for personal simulation training (Gather, Reaction, Discuss clinical component, Analysis with deepening, Summary, Application / Transfer to Reality), and Observe could be used.

3. Micro-components for online staged debriefing are short explanations and comments on positive and negative learner actions in microformat as text, links, short videos, audios, images, quizzes, and dialogue simulators integrated into simulation training.

4. Simulation training can be divided into logical blocks depending on the steps in medical care and the patient's way (with multi-location simulation) or the components of medical care provision.

5. Staged debriefing can be integrated into the structure of online simulation training and happen simultaneously with a clinical case scenario, as micro-debriefing, or can be carried out within the simulation training synchronously with the training scenario, asynchronously with the training group, or synchronously in collaborative learning.

6. Staged debriefing with defragmentation may be considered for complex clinical scenarios with multiple decision points or multi-location simulation when the goals and list of competencies of each location are different, aiming not to transfer all medical errors to the end of the clinical scenario and mastering the process in steps.

7. Pre-scripting could be helpful in the planning of simulation training [33] and in the defragmented debriefing model as well.

8. Stratification of medical errors can be used to identify decision points in a simulation and predict how events will unfold as well as generate feedback or cognitive support needed at each stage.

9. When preparing a simulation training script, it is necessary to combine the main blocks of the script with micro-components of debriefing into a single logical sequence, but with opportunities to build adaptive learning and branching development of events through adding conditions.

10. The final formula of any training is individual and determined by the training goals and the set of competencies acquired during the training.

11. A staged defragmented debriefing model as integrated micro-debriefing components inside simulation can be considered for training for competencies formation and could be helpful in assessing specific cases.

12. A staged defragmented debriefing model as integrated micro-debriefing components inside simulation can inform both online and offline training.

13. Branching technology as an effective learning strategy [34,35] can be used to implement multiple repetitions within individual blocks in a defragmented debriefing model to fully master competencies so that errors do not accumulate and are not transferred to the end of the simulation scenario.

14. An adaptive training format is recommended when conducting debriefing in which certain micro-components of the debriefing are given to the student only when necessary, depending on the student’s actions.

15. Conducting a staged defragmented debriefing model as integrated micro-debriefing components inside online simulation can have benefits for simulation training.

16. When using this debriefing model, standardization of debriefing and obtaining competencies, automated facilitation, and a change in the role of the teacher are achieved.

17. Employing a staged debriefing approach involving micro-components integrated into simulation training empowers students by granting them autonomy both from fellow participants and the instructor.

18. This arrangement allows for the immediate reception of feedback and assistance during simulation training, which proves invaluable when navigating complex scenarios that demand multifaceted clinical reasoning, rather than simple, one-dimensional solutions. This approach aids in averting the accumulation of errors toward the conclusion of the simulation training and gaining clinical experience.

Similar to how molecules consist of atoms, simulation training and debriefing comprise different elements. Just as molecules comprise different types of atoms, debriefing can be dissected into its constituent components and seamlessly integrated into clinical scenarios to enhance simulation results.

Participants who experienced simulation training with the staged defragmented debriefing model consistently rated their clinical decision-making skills higher compared to those in the control group receiving traditional debriefing (4.15 ± 0.59 vs. 3.55 ± 0.51; p-value = 0.002). This suggests that the staged debriefing model contributed to a clearer and more effective decision-making process during the simulation scenarios.

Moreover, participants in the staged defragmented debriefing model group reported greater competencies formation compared to the traditional debriefing group (4.3 ± 0.47 vs. 3.55 ± 0.51; p-value < 0.001), indicating the model's efficacy in fostering critical competencies essential for clinical practice.

Regarding knowledge retention, participants in the staged defragmented debriefing model group demonstrated better retention of key concepts and information compared to the traditional debriefing group (4.35 ± 0.49 vs. 3.44 ± 0.51; p-value < 0.001), highlighting the model's effectiveness in facilitating long-term learning outcomes.

Additionally, feedback quality was perceived to be significantly higher in the staged defragmented debriefing model group compared to the traditional debriefing group (4.50 ± 0.51 vs. 4.11 ± 0.58; p-value =0.03), indicating greater satisfaction with the depth and relevance of feedback provided during the debriefing sessions.

Participants in the traditional debriefing group reported higher levels of engagement during debriefing sessions compared to the staged defragmented debriefing model group (4.83 ± 0.38 vs. 4.40 ± 0.50; p-value = 0.005) and not significant as more relevant by participants (4.61 ± 0.50 vs. 4.35 ± 0.49; p>0.05).

Overall, participants reported a more positive and enriching learning experience in the staged defragmented debriefing model group compared to the traditional debriefing group (4.45 ± 0.51 vs. 4.05 ± 0.64; p-value = 0.04), highlighting the model's effectiveness in enhancing the overall learning experience.

There were no significant differences between the staged defragmented debriefing model group and the traditional debriefing group based on the impact on clinical practice (4.20 ± 0.62 vs. 4.00 ± 0.48; p-value > 0.05) and comparison with expectations (4.20 ± 0.41 vs. 4.05 ± 0.73; p-value > 0.05).

## Discussion

The classical structure of simulation training typically follows a step-by-step process that includes pre-briefing, scenario introduction, simulation, debriefing, reflection and feedback, and post-simulation learning [36-39].

Debriefing after simulation-based learning for healthcare students leads to a significant increase in confidence in their ability to care for unstable patients [40-43]. Debriefing should be based on a structured framework; however, researchers differ as to which framework is most effective [21,22]. In health care, numerous debriefing techniques and models have been suggested and put into practice. However, a universally recognized gold standard for debriefing has yet to be identified [27].

Debriefing can take place in various formats such as structured debriefing models (e.g., Plus-Delta, Three-Phase Debriefing Technique [reaction, analysis, and summary or RAS]), multiphase debriefing techniques: Promoting Excellence And Reflective Learning in Simulation (PEARLS), team-guided self-correction, Advocacy-Inquiry, and Systemic-constructivist (TeamGAINS), health care simulation After-Action Review (AAR), guided reflection, or facilitated group discussion. The choice of the debriefing method depends on the learning objectives, participant characteristics, and simulation scenario [20-28,44-46].

Effective debriefing in clinical simulation training promotes active learning, self-reflection, and skill enhancement. It helps bridge the gap between simulation experiences and real-world clinical practice, fostering the transfer of knowledge and skills to patient care settings [5].

The multicenter, cluster-randomized, controlled trial provided debriefing scripts to faculty, facilitating simulated pediatric resuscitation scenarios and improving debriefing quality, especially for novices and those at large sites. The debriefing script may improve the facilitator’s ability to lead the debrief effectively and may enhance knowledge acquisition [33]. To elaborate, debriefings conducted after cardiac arrest resuscitations have shown associations with enhanced adherence to resuscitation protocols, increased patient survival rates, and improved neurological outcomes [47-50].

Numerous simulation programs have recently transitioned to offering remote simulations coupled with virtual debriefing sessions. In this context, virtual debriefings involve educators guiding discussions via online video conferencing platforms [13-19].

Debriefing in virtual simulation can take several forms: in-person (on-site), self-debriefing, and synchronous or asynchronous debriefing [18,19]. The physical presence of the trainers and protagonists is not mandatory for digital debriefing [21].

Beyond the conventional on-site simulation training debriefing approach, viable alternatives to debriefing virtual medical simulations in education include synchronous virtual debriefing through video conferencing platforms, asynchronous debriefing via online discussion boards, computer-based debriefing, and self-debriefing.

All debriefing methods demonstrated some benefits, and the debriefer’s experience level was also important. Additional research is needed to determine the most effective methods to support different levels of learners [16].

The debriefing models outlined may not align perfectly with the context of online simulation training integrated with automated debriefing. Presently, the debriefing approaches to online simulation training closely resemble traditional models, necessitating post-simulation group discussions and teacher training. However, incorporating debriefing elements directly into the structure of simulation training diminishes reliance on the trainer's expertise, granting students greater autonomy. This integration has the potential to enhance the quality of simulation training and promote student independence. Without face-to-face interactions or immediate feedback, some learners may struggle to stay engaged, leading to decreased participation and less successful learning outcomes. Conducting a standardized online debriefing requires special advance preparation. Nevertheless, the anticipated benefits and effectiveness are expected to be considerably high.

Nonetheless, it is important to address and overcome these challenges through the implementation of new debriefing strategies and techniques for effective debriefing. By addressing these issues, the benefits of clinical debriefing in simulation training can be maximized, leading to enhanced learning, skill development, and patient care outcomes.

Presently, it is particularly important to maintain consistent quality in simulation training while also automating the debriefing process, even during asynchronous individual training.

In the rapidly evolving landscape of simulation training, maintaining consistent quality is crucial to ensure effective learning outcomes. Automation of the debriefing process, especially during asynchronous individual training, becomes essential for several reasons. Firstly, it allows for timely and personalized feedback, enabling learners to reflect on their performance promptly. Secondly, it enhances accessibility, making debriefing available to learners at their convenience. Lastly, consistent quality in debriefing is vital for standardizing the learning experience and promoting uniform skill development across diverse training scenarios.

Simultaneously, the outcomes of simulation training are intricately linked to the caliber of the debriefing [5]. The incorporation of a standardized model has the potential to standardize the quality of outcomes, simplify the facilitator’s role, and detach the results of simulation training from the trainer’s individual readiness and subject mastery.

This training structure empowers students to independently complete their training, irrespective of their training group or instructor. This independence is particularly vital in the context of online asynchronous learning. In this format, the role of the training group leader or simulation training coordinator primarily revolves around resolving organizational matters and analyzing and generating completion reports about the simulation training.

The reflective component of debriefing is indeed a crucial element, and the presented new model of online debriefing is designed to effectively anchor experiential learning theory through the automated provision of information for comprehension and reflection. In the proposed debriefing model, information for comprehension and reflection is automatically provided based on the student’s actions. In proposed debriefing model strategically integrates a matrix of potential medical errors for each specific case, and closely aligns with the model of stratification of medical errors. This matrix serves as the foundation for calculating reflection points, allowing for a targeted and meaningful reflective process. By automating the provision of relevant information for comprehension and reflection, the proposed model ensures that learners engage in a thoughtful analysis of their actions and decisions during the simulated scenario. Embedding this automated reflective component into this online debriefing model was aimed to facilitate a more comprehensive and meaningful learning experience for participants, promoting deeper engagement with the simulation and more effective integration of experiential learning theory.

The anticipated advantages of implementing a staged defragmented debriefing model as integrated micro-debriefing components inside online simulation include immediate feedback, adaptive learning format, micro-learning, guided simulation, decision-making process during the patient‘s way with specific nosology, the step-by-step decision-making process in clinical situations, nonaccumulation of errors, nontransference of errors to the end of the scenario (they are worked out in parts), demonstration of clinical decision-making algorithms, deep cognitive simulation, deep memorization, experience gained as in real clinical practice, quick acquisition of medical experience, and facilitating the work of the trainer, because debriefing is integrated into the structure of simulation training and is carried out automatically.

In essence, the goal is to ensure that when a chosen debriefing model is employed, it provides a consistent and standardized educational experience for all participants, regardless of their geographical location or institutional affiliation in the particular online clinical simulation. This approach seeks to enhance the reliability and comparability of educational outcomes within the specific context of the concert simulation training (scenario).

This approach does not assert that an effective online debriefing model must be entirely independent of the trainer(s). However, this strategy recognizes that the landscape of online learning provides unique opportunities for learner independence, and the role of the trainer is evolving. While acknowledging the importance of the trainer's role, the suggested approach also emphasizes leveraging online learning opportunities to empower trainees with a certain degree of independence. This does not imply eliminating the trainer's involvement but rather redefining their role to foster a more learner-centric and self-directed approach. The goal is to design a debriefing model that capitalizes on the strengths of online learning, allowing trainees to take a more active role in their learning process. This shift toward greater independence aligns with the evolving nature of education in virtual environments. The objective is to facilitate a learning experience that encourages autonomy while ensuring the quality of simulation training is maintained.

The effectiveness of microlearning has long been globally proven and is beyond doubt. Surprisingly, this approach has not been previously utilized for debriefing. This approach enables to address of a large number of medical errors incrementally throughout the simulation training, rather than deferring them to the end. This proves advantageous for serious games. For instance, a patient with acute myocardial infarction receives care across different stages (paramedic team, catheter laboratory, intensive care ward, general ward, follow-up). These locations present diverse simulation settings, each with distinct competencies and learning objectives. Transferring medical errors from the paramedic stage to the conclusion of the simulation training is impractical. Based on this approach it is possible to construct multi-location simulation training that mirrors a patient's journey in real clinical practice. This strategy could ensure the high-quality acquisition of competencies.

The staged defragmented debriefing model involves not only providing feedback on specific actions but also guiding learners through a step-by-step decision-making process, demonstrating clinical decision-making algorithms, and promoting deep cognitive simulation. It combines elements traditionally associated with both debriefing and feedback to create a holistic learning experience.

The defragmented developed debriefing model could be the preferred teaching strategy for competency formation based on the author's point of view.

A limitation of this study is the need for external validation of the proposed debriefing model through pedagogical experiments in the context of medical simulation. Although the model shows promise, its effectiveness and applicability should be further tested and validated. It remains unclear whether this debriefing model, primarily designed for online simulation training, can be seamlessly adapted to on-site simulation training scenarios.

It is important to acknowledge the potential risk of cognitive overload for students when implementing the proposed model. The integration of micro-debriefing components at various points in simulation scenarios may require students to process a significant amount of information simultaneously, which could lead to cognitive overload. Strategies to mitigate this risk may include structuring the debriefing process to focus on key learning objectives and providing adequate breaks between simulation scenarios to allow for reflection and consolidation of learning.

Another challenge to consider is the need to prepare and educate trainers and clinical case scriptwriters to conduct the proposed debriefing model effectively. Trainers and clinical scriptwriters may require specialized training to facilitate a defragmented debriefing model.

A potential perspective for enhancing the feasibility and scalability of the proposed model is to explore the automation of scenario generation with this debriefing model for integrating microdebriefing components into simulation scenarios. By automating aspects of the clinical scenarios creation the burden on individuals responsible for preparing simulation scenarios may be reduced.

Future researchers should explore the model’s versatility and potential modifications required for on-site simulation training and online simulation.

## Conclusions

One of the advantages of online clinical simulation is its flexibility and accessibility. Participants can engage in simulation activities remotely at their own pace and convenience without being physically present in a simulation center or clinical setting.

Debriefing is a fundamental pillar of successful simulation-based medical education. Although universally accepted, evidence-based gold standard debriefing technique remains undiscovered. It is probable that many of these debriefing approaches yield positive outcomes when employed correctly by educators and facilitators.

There is a need for enhancements in online debriefing models. Staged debriefing promotes reflection, critical thinking, and self-assessment, ultimately enhancing participants’ learning and performance in clinical practice.

This work highlights the imperative need for advancements in online debriefing models. The introduction of a staged defragmented debriefing model, with integrated micro-debriefing components, presents a novel avenue for promoting reflection, critical thinking, and self-assessment in participants. The model, designed to accommodate the flexibility of online learning, could contribute to heightened learning and performance in clinical practice. This model does not preclude the option of a traditional debriefing with an instructor. However, in the absence of an instructor, it still ensures the attainment of competency at an appropriate level. This prompts essential considerations about the extent to which the model can independently debrief learners online and under what circumstances human instructor involvement may be necessary.

As key take-home messages, this work underscores the potential of the staged debriefing model with micro-debriefing components to enrich the learning experience in online clinical simulation.

## Appendices

An example of clinical case scenario defragmentation in multilocation simulation: clinical case scenario “A Lady with a sudden cardiac pain”

The unblinded title “Acute myocardial infarction with ST-segment elevation due to spontaneous coronary dissection”

Discussed topics in the clinical cases simulator:

Acute coronary syndrome, an electrocardiogram (ECG), myocardial infarction, myocardial infarction at a young age, spontaneous coronary artery dissection (SCAD), myocardial infarction with non-obstructive coronary arteries, and fibromuscular dysplasia.

Clinical guidelines discussed in clinical cases:

1. Spontaneous Coronary Artery Dissection: Current State of the Science. A Scientific Statement From the American Heart Association. 2018.

2. 2017 ESC Guidelines for the management of acute myocardial infarction in patients presenting with ST-segment elevation: The Task Force for the management of acute myocardial infarction in patients presenting with ST-segment elevation of the European Society of Cardiology (ESC).

3. ESC working group position paper on myocardial infarction with non-obstructive coronary arteries.

4. European Society of Cardiology, Acute Cardiovascular Care Association, SCAD study group: a position paper on spontaneous coronary artery dissection.

5. First International Consensus on the diagnosis and management of fibromuscular dysplasia.

6. Fourth universal definition of myocardial infarction (2018).

7. The Maryland Medical Protocols for Emergency Medical Services Providers

The general structure of the training:

· Communication with the patient in dialogue simulators;

· Objective examination of a patient who has a sudden pain in the heart;

· Carrying out the necessary additional examinations (registration and interpretation of the ECG);

· Working with the paramedic team, thus providing first aid to a patient with acute coronary syndrome at the site of the incident;

· Training in the assistance algorithm of the paramedic team for a patient with acute coronary syndrome;

· Looking through the causes of intense pain in the heart; causes of acute coronary syndrome;

· Checking understanding of the results of coronary angiography;

· Analyzing the results of echocardiography;

· Taking part in the daily rounds (intensive care unit and general ward);

· Analyzing international recommendations and available international experience from the standpoint of a clinical case;

· Taking part in the consultations and choosing the most optimal treatment tactics;

· Assessing the patient’s condition in dynamics during the stay in hospital;

· Look through the general algorithm for the rehabilitation of patients and choose the most optimal rehabilitation mode for the patient, taking into account the peculiarities of the clinical situation;

· Providing post-observation of the patient at the outpatient stage with an in-depth follow-up examination and analysis of the condition in dynamics;

· Providing therapy correction on an outpatient basis;

· Myocardial injury markers, injury markers rise time, injury assessment;

· Types of acute myocardial infarction;

· Causes of myocardial infarction (embolism of the epicardial coronary arteries, myocardial "bridges", thrombophilia, MINOCA, Takotsubo syndrome, artery abnormalities, hypercoagulation, epicardial spasm, and microvascular spasm);

· Anatomy of coronary artery dissection;

· NHLBI dissection classification;

· Saw SCAD classification;

· TIMI blood flow assessment;

· Guidelines-based management of acute spontaneous coronary artery dissection;

· The causes of spontaneous coronary artery dissection in the population (injury, fibromuscular dysplasia, pregnancy, childbirth, congenital heart disease, hormones usage, congenital arteriopathies, autoimmune connective tissue disorders) and triggers identification;

· Anatomical basis of fibromuscular dysplasia as an etiology factor of the SCAD;

· Focal or multifocal vascular fibromuscular dysplasia;

· Echocardiography in the patient with acute myocardial infarction;

· Rehabilitation program for patients with acute myocardial infarction;

· The algorithm for risk stratification for patients with acute myocardial infarction for mobilization;

· Evaluation of Rehabilitation tests;

· General scheme of rehabilitation in patients with acute myocardial infarction;

· Contraindications to physical activity in the schemes of rehabilitation of patients after acute myocardial infarction;

· Rehabilitation for patients with SCAD after discharge from the hospital;

· Drug therapy in patients after myocardial infarction due to SCAD.

**Table 6:** The example of clinical case scenario defragmentation in multilocation simulation: clinical case scenario “A Lady with a sudden cardiac pain” a: analysis (with deepening); a/t: application and transfer to reality; g: gather; d: description phase; r: reaction and report; dcc: discuss clinical component; o: observe the patient's state; s: summary. CABG: a coronary artery bypass graft; CT: computed tomography; ECG: electrocardiogram; ESC: European Society of Cardiology; ICU: Intensive Care Unit; MINOCA: myocardial infarction with nonobstructive coronary arteries; NHLBI: National Heart, Lung, and Blood Institute; PCI: percutaneous coronary intervention; SCAD: spontaneous coronary artery dissection; STEMI: ST-Segment Elevation Myocardial Infarction; TIMI: The Thrombolysis in Myocardial Infarction.

Location	Description
1 location “A”	*Title: *Introduction. *Content:* video presentation of the head physician of the virtual clinic – Briefing. *Topics:* adaptation for simulation online training. *Competences:* -
2 location “B”	*Title:* Paramedics work.* Content: *Dialogue with the patient; Objective examination; ECG registration; ECG interpretation with test control; J-point theory; J-point finding test; Step medical care prehospital algorithm for myocardial infarction for ST-segment elevation; Paramedics dialogue with PCI center. *Topics: *J-point on ECG; Criteria of myocardial infarction with ST-segment elevation (to meet criteria or not); Step medical care prehospital algorithm for myocardial infarction for ST-segment elevation; Information that should be discussed during paramedics' call to the PCI center. *Competences: *Communication with a patient during first medical care (if communication is with empathy, the right set of questions, then without debriefing intervention, if communication is without empathy and not by complaints and the logic of the survey, debriefing intervention is possible in the next formats - *discuss clinical component (dcc), observe the patient's state (o); *Objective examination; Placing electrodes for ECG registration *(if incorrect - debriefing intervention and repeating the procedure until correct execution in the next formats - discuss clinical component (dcc);* J-point finding on the ECG *(if incorrect -debriefing intervention and repeating the procedure until correct execution in the next formats - discuss clinical component (dcc), analysis (a)); *Establishing criteria for myocardial infarction with ST-segment elevation by ECG *(if incorrect -debriefing intervention and repeating the procedure until correct execution in the next formats - discuss clinical component (dcc), analysis (a)); *Mastering of prehospital algorithm for myocardial infarction for ST-segment elevation (if incorrect -debriefing intervention and repeating the procedure until correct execution in the next formats -* discuss clinical component (dcc), analysis (a))*; Communication with PCI center, choosing priority for hospitalization *(if the patient’s presentation to the reperfusion center is correct, then without debriefing intervention, if incorrect - debriefing intervention and repeating the call to the reperfusion center until it is completed correctly in the next formats - discuss clinical component (dcc)).*
3 location “C”	*Title: *Patient transportation to the hospital. *Content:* Delays in the medical care of acute coronary syndrome patients with ST-segment elevation (STEMI); Choosing the patient's transportation scheme and medical care based on the distance to the hospital; *Topics:* Delays in the medical care of acute coronary syndrome patients with ST-segment elevation (STEMI). Competences: Choosing the patient's transportation scheme and medical care based on the distance to the hospital *(if incorrect - debriefing intervention and repeating the procedure until correct execution in the next formats - discuss clinical component (dcc), analysis (a));*
4 location “D”	*Title: *Hospital emergency room. *Content:* Repeat ECG, interpretation in dynamics; Blood tests chosen at the admission department stage; Assessment of patient’s quick blood test results; Choosing the location for patient transferring after admission department; Myocardial injury markers, injury markers rise time, injury assessment; *Topics:* Myocardial injury markers, injury markers rise time, injury assessment. *Competences: *Repeat ECG evaluation, interpretation in dynamics *(if incorrect -debriefing intervention and repeating the procedure until correct execution in the next formats - discuss clinical component (dcc), analysis (a));* Blood tests chosen at admission department stage *(if incorrect -debriefing intervention and repeating the procedure until correct execution in the next formats - discuss clinical component (dcc));* Assessment of patient’s quick blood tests results *(if incorrect -debriefing intervention and repeating the procedure until correct execution in the next formats - discuss clinical component (dcc), analysis (a));* Choosing the location for patient transferring after admission department *(if incorrect -debriefing intervention and repeating the procedure until correct execution in the next formats - discuss clinical component (dcc), analysis (a)).*
5 location “E”	*Title:* Colleagues communication.* Content:* Types of acute myocardial infarction; Making hypotheses of the causes of myocardial infarction in a young person (embolism of the epicardial coronary arteries, myocardial "bridges", thrombophilia, MINOCA, Takotsubo syndrome, artery abnormalities, hypercoagulation, epicardial spasm, and microvascular spasm) during communication; Understanding of different types of myocardial infarction. *Topics:* Types of acute myocardial infarction; Causes of myocardial infarction (embolism of the epicardial coronary arteries, myocardial "bridges", thrombophilia, MINOCA, Takotsubo syndrome, artery abnormalities. *Competences: *Matching different types of myocardial infarction with their essence *(if incorrect -debriefing intervention and repeating the procedure until correct execution in the next formats - discuss clinical component (dcc), analysis (a)).*
6 location “F”	*Title: *Catheter Laboratory. *Content:* Watching the patient's coronary angiography results (video); Choosing the treatment strategy in this clinical case based on the revealed changes detected during coronary angiography (demonstration of the subsequence of crucial decision for the discussed clinical case); Assessment of the understanding of the obtained data during coronary angiography (work with images); Understanding the anatomical nature of coronary artery dissection; NHLBI score for coronary artery dissection in a patient; Evaluation of a patient's coronary artery dissection according to the Saw classification; TIMI assessment of patient's coronary artery blood flow; Discussion of a patient management strategy based on international guidelines for spontaneous dissection of coronary arteries; Discussing management of acute spontaneous coronary artery dissection. *Topics: *Anatomy of coronary artery dissection; NHLBI dissection classification; Saw SCAD classification; TIMI blood flow assessment; Guidelines-based management of acute spontaneous coronary artery dissection. *Competences:* Interpretation of patient's coronary angiography data *(if incorrect -debriefing intervention and repeating the procedure until correct execution in the next formats -* *discuss clinical component (dcc), analysis (a))*; Assessment of patient's coronary angiography results based on NHLBI score for coronary artery dissection in a patient. *(if incorrect -debriefing intervention and repeating the procedure until correct execution in the next formats - discuss clinical component (dcc), analysis (a)); *Evaluation of patient's coronary angiography results based on the Saw classification for coronary artery dissection. *(if incorrect -debriefing intervention and repeating the procedure until correct execution in the next formats - discuss clinical component (dcc), analysis (a));* Assessment of the patient's blood flow according to the TIMI scale. *(if incorrect -debriefing intervention and repeating the procedure until correct execution in the next formats - discuss clinical component (dcc), analysis (a)); *Choosing the treatment strategy based on the identified changes in the coronary arteries and international clinical guidelines in a particular clinical case. *(if incorrect -debriefing intervention and repeating the procedure until correct execution in the next formats - discuss clinical component (dcc), analysis (a), observe the patient's state (o), “+” or ) “-” depending on which strategy was chosen, with stenting or CABG the patient’s condition worsens and potentially leads to death; if drug therapy is chosen, the patient’s condition improves.*
7 location “G”	*Title: *Consultation. *Content: *Analysis of the causes of spontaneous coronary artery dissection in the population (injury, fibromuscular dysplasia, pregnancy, childbirth, congenital heart disease, hormones usage, congenital arteriopathies, autoimmune connective tissue disorders) and triggers identification; Understanding the anatomical basis of fibromuscular dysplasia as an etiology factor of the SCAD; Understanding the terms “Focal or multifocal vascular fibromuscular dysplasia”. *Topics:* The causes of spontaneous coronary artery dissection in the population (injury, fibromuscular dysplasia, pregnancy, childbirth, congenital heart disease, hormone usage, congenital arteriopathies, autoimmune connective tissue disorders) and triggers identification; Anatomical basis of fibromuscular dysplasia as an etiology factor of the SCAD; Focal or multifocal vascular fibromuscular dysplasia. *Competences: *Choosing the pathologies for differential diagnosis of SCAD etiology* (if incorrect -debriefing intervention and repeating the procedure until correct execution in the next formats - discuss clinical component (dcc), analysis (a)).*
8 location “H”	*Title:* Intensive care unit. *Content: *Dialogue with the patient in the intensive care unit after coronary angiography and understanding the underlying causes of SCAD; Objective examination of the patient at ICU; Analyzing the results of laboratory tests; Patient's ECG interpretation at ICU; Comparing patient's ECG at ICU and admission department; Watching the patient's echocardiography; Interpretation of patient's echocardiography; Prescribing therapy to the patient based on revealed data and international guidelines; Summing up information about the patient. *Topics: *Echocardiography in the patient with acute myocardial infarction. *Competences: *Communication with the patient with acute myocardial infarction and SCAD in the intensive care unit *(if communication is with empathy, the right set of questions, then without debriefing intervention, if communication is without empathy and not in accordance with complaints and the logic of the survey, debriefing intervention is possible in the next formats - discuss clinical component (dcc), observe the patient's state (o); *Performing objective examination of the patient at the ICU; Analyzing the results of laboratory tests *(if incorrect -debriefing intervention and repeating the procedure until correct execution in the next formats - discuss clinical component (dcc), analysis (a)); *Patient's ECG interpretation at the ICU *(if incorrect -debriefing intervention and repeating the procedure until correct execution in the next formats - discuss clinical component (dcc), analysis (a));* Comparing patient's ECG at ICU and admission department *(if incorrect -debriefing intervention and repeating the procedure until correct execution in the next formats - discuss clinical component (dcc), analysis (a)); *Interpretation of patient's echocardiography *(if incorrect -debriefing intervention and repeating the procedure until correct execution in the next formats - discuss clinical component (dcc), analysis (a)); *Prescribing therapy to the patient based on revealed data and international guidelines *(if incorrect -debriefing intervention and repeating the procedure until correct execution in the next formats - discuss clinical component (dcc), analysis (a));* Summing up information about the patient after diagnostic tests *(if incorrect -debriefing intervention and repeating the procedure until correct execution in the next formats - discuss clinical component (dcc), analysis (a)).*
9 locations “I”	*Title:* CT Scan room. Content: CT in patients with SCAD.* Topics*: -. *Competences: *Understanding the results of CT *(if incorrect -debriefing intervention and repeating the procedure until correct execution in the next formats - discuss clinical component (dcc), analysis (a)).*
10 location “J”	*Title: *General ward. *Content: *The dialogue with the patient with acute myocardial infarction in the general ward during the hospitalization period; Objective examination in the general ward during the hospitalization period; ECG day 2, ECG day 3, ECG day 5, ECG day 10 interpretation; Comparing ECG day 2, ECG day 3, ECG day 5, and ECG day 10 with the ECG day 1 acute myocardial infarction; Assessment of the result of blood tests during the period of hospitalization, identification of possible complications; Choosing a rehabilitation program for the patient in the particular clinical case: Rehabilitation program for patients with acute myocardial infarction; The algorithm for risk stratification for patients with acute myocardial infarction for mobilization; Evaluation of Rehabilitation tests; General scheme of rehabilitation in patients with acute myocardial infarction; Contraindications to physical activity in the schemes of rehabilitation of patients after acute myocardial infarction. *Topics:* Rehabilitation program for patients with acute myocardial infarction; The algorithm for risk stratification for patients with acute myocardial infarction for mobilization; Evaluation of Rehabilitation tests; General scheme of rehabilitation in patients with acute myocardial infarction; Contraindications to physical activity in the schemes of rehabilitation of patients after acute myocardial infarction. *Competences:* Communication with the patient with acute myocardial infarction in the general ward during the hospitalization period *(if communication is with empathy, the right set of questions, then without debriefing intervention, if communication is without empathy and not by complaints and the logic of the survey, debriefing intervention is possible in the next formats - discuss clinical component (dcc), observe the patient's state (o);* Objective examination in the general ward during the hospitalization period; ECG day 2, ECG day 3, ECG day 5, ECG day 10 interpretation *(if incorrect -debriefing intervention and repeating the procedure until correct execution in the next formats - discuss clinical component (dcc), analysis (a));* Comparing ECG day 2, ECG day 3, ECG day 5, and ECG day 10 with the ECG day 1 acute myocardial infarction *(if incorrect -debriefing intervention and repeating the procedure until correct execution in the next formats - discuss clinical component (dcc), analysis (a)); *Assessment of the result of blood tests during the period of hospitalization, identification of possible complications *(if incorrect -debriefing intervention and repeating the procedure until correct execution in the next formats - discuss clinical component (dcc), analysis (a)); *Choosing a rehabilitation program for the patient in the particular clinical case *(if incorrect -debriefing intervention and repeating the procedure until correct execution in the next formats - discuss clinical component (dcc), analysis (a)).*
11 location “K”	*Title: *Consultation before discharge. *Content: *Summing up all periods of hospitalization, patient state, and data of diagnostic tests with rationale treatment strategy in the discussed clinical case. Making a final diagnosis in this clinical case. Discussing further rehabilitation for patients with SCAD after discharge from the hospital.* Topics: *Rehabilitation for patients with SCAD after discharge from the hospital. *Competences:* Making a final diagnosis in this clinical case *(if incorrect -debriefing intervention and repeating the procedure until correct execution in the next formats - discuss clinical component (dcc), analysis (a)).*
12 location “L”	*Title: *Follow-up in a patient with SCAD. *Content: *Dialogue with a patient during follow-up; An objective examination of the patient during follow-up; ECG interpretation after six weeks after discharge; Comparing the ECG day ten during hospitalization with ECG after six weeks after discharge; Evaluation of the results of repeat coronary angiography after six weeks after discharge; Evaluation of laboratory data after six weeks after discharge; Prescribing and correction of drug therapy. *Topics:* Drug therapy in patients after myocardial infarction due to SCAD. *Competences: *Communication with the patient during follow-up *(if communication is with empathy, the right set of questions, then without debriefing intervention, if communication is without empathy and not by complaints and the logic of the survey, debriefing intervention is possible in the next formats - discuss clinical component (dcc), observe the patient's state (o); *An objective examination of the patient during follow-up *(if incorrect -debriefing intervention and repeating the procedure until correct execution in the next formats - discuss clinical component (dcc), analysis (a));* ECG interpretation after six weeks after discharge *(if incorrect -debriefing intervention and repeating the procedure until correct execution in the next formats - discuss clinical component (dcc), analysis (a));* Comparing the ECG day ten during hospitalization with ECG six weeks after discharge *(if incorrect -debriefing intervention and repeating the procedure until correct execution in the next formats - discuss clinical component (dcc), analysis (a));* Evaluation of the results of repeat coronary angiography six weeks after discharge *(if incorrect -debriefing intervention and repeating the procedure until correct execution in the next formats - discuss clinical component (dcc), analysis (a)); *Evaluation of laboratory data six weeks after discharge* (if incorrect -debriefing intervention and repeating the procedure until correct execution in the next formats - discuss clinical component (dcc), analysis (a)); *Prescribing and correction of drug therapy during patient’s follow-up *(if incorrect -debriefing intervention and repeating the procedure until correct execution in the next formats - discuss clinical component (dcc), analysis (a)).*
13 location “M”	*Title: *Feedback on training. *Content:* Six questions to the trainee for assessment of the simulation training. *Topics:* -. *Competences: *Summary (s); Description Phase (d); Reaction (r).
14 location “N”	*Title: *Summing up. *Content:* General test on all locations of clinical case simulator - 30 groups of questions, 164 questions, 30 random questions to each trainee. *Topics:* -. *Competences:* Summing up the data of the clinical case simulator “A Lady with a sudden cardiac pain” (summary (s), application/transfer to reality (a/t)).
15 location “O”	*Title:* Recommended literature. *Content: *The list of literature with links. *Topics: *Spontaneous Coronary Artery Dissection; Acute myocardial infarction in patients presenting with ST-segment elevation; Myocardial infarction with non-obstructive coronary arteries; Diagnosis and management of fibromuscular dysplasia; Emergency Medical Protocols.* Competences: *Understanding of international clinical guidelines and providing diagnostic and treatment in particular clinical situations based on evidence-based medicine (summary (s); application/transfer to reality (a/t)).
16 location “P”	*Title: *Case simulator discussion (group discussion – asynchronous board, chat, or synchronous - webinar). *Content: *Communication between participants of the training. *Topics: *Communication between participants of training devoted to presented clinical cases, similar clinical cases, and the topics discussed in the clinical case simulator. *Competences: *Communications with other participants of training (social learning) in virtual discussions, trainer feedback (summary (s); application/transfer to reality (a/t); gather (g); description phase (d); reaction and report (r); discuss clinical component (dcc)).

With the right strategy, encouraging phrases that support the choice of the right strategy are also important and actively used.
